# Evolution of Carbon Ion Radiotherapy at the National Institute of Radiological Sciences in Japan

**DOI:** 10.3390/cancers10030066

**Published:** 2018-03-06

**Authors:** Osama Mohamad, Hirokazu Makishima, Tadashi Kamada

**Affiliations:** 1 Hospital of the National Institute of Radiological Sciences, National Institutes for Quantum and Radiological Science and Technology, 4-9-1 Anagawa, Inage-ku, Chiba 263-8555, Japan; Osama.mohamad@utsouthwestern.edu (O.M.); makishima.hirokazu@qst.go.jp (H.M.); 2 Department of Radiation Oncology, University of Texas—Southwestern Medical Center, 2280 Inwood Rd., Dallas, TX 75390, USA

**Keywords:** carbon ion radiotherapy, CIRT, National Institute of Radiological Sciences, NIRS, particle therapy

## Abstract

Charged particles can achieve better dose distribution and higher biological effectiveness compared to photon radiotherapy. Carbon ions are considered an optimal candidate for cancer treatment using particles. The National Institute of Radiological Sciences (NIRS) in Chiba, Japan was the first radiotherapy hospital dedicated for carbon ion treatments in the world. Since its establishment in 1994, the NIRS has pioneered this therapy with more than 69 clinical trials so far, and hundreds of ancillary projects in physics and radiobiology. In this review, we will discuss the evolution of carbon ion radiotherapy at the NIRS and some of the current and future projects in the field.

## 1. Introduction

Particle therapy (PT), especially with heavy ions such as carbon, is an attractive radiation modality with significant physical and biological advantages over photon irradiation and deserves special attention with respect to patient selection, treatment planning, and delivery. Since the middle of the twentieth century, however, particle therapy has been the subject of considerable debate. There is a consensus among radiation oncologists, physicists, and radiobiologists that heavy particles have superior physical and biological properties over photons [1,2,3]. The main debate, however, has always been whether these physical and biological advantages translate into clinical value to justify the high cost of construction and maintenance of PT centers and the increased treatment cost. This debate now continues with strong opinions on both sides especially in the wider context of the socio-economic status of health-care delivery.

Unfortunately, little data comparing PT to photon radiation has been produced over the past decades. Despite the lack of any phase III randomized trials data, the so-far reported oncologic outcomes and side effect profiles of carbon ion radiotherapy (CIRT) are very encouraging [3,4]. Some critics argue, however, that phase III randomized data are not needed to justify the adoption of PT. In either case, more efficacy data is needed. When combined with cost-conscious patient selection and shorter treatments (hypofractionation), such data may establish the cost-effectiveness of this radiation modality [5,6,7]. We need to better understand the radiobiology and physics behind CIRT and critically analyze the emerging data to improve the design of advanced clinical trials and engineering of the next generation machines.

The Heavy Ion Medical Accelerator in Chiba (HIMAC) was built by the National Institute of Radiological Sciences (NIRS) to be the first heavy ion medical accelerator system specifically dedicated for clinical research and patient care. Since its inception, the HIMAC has adopted a patient-centered approach to treating patients with cancer while maintaining a safe environment for its staff. In this review, the history of CIRT at the NIRS, the evolution of the technology and clinical practice, and the future advances in the field will be discussed.

## 2. A Brief History of the National Institute of Radiological Sciences

**Figure 1:**
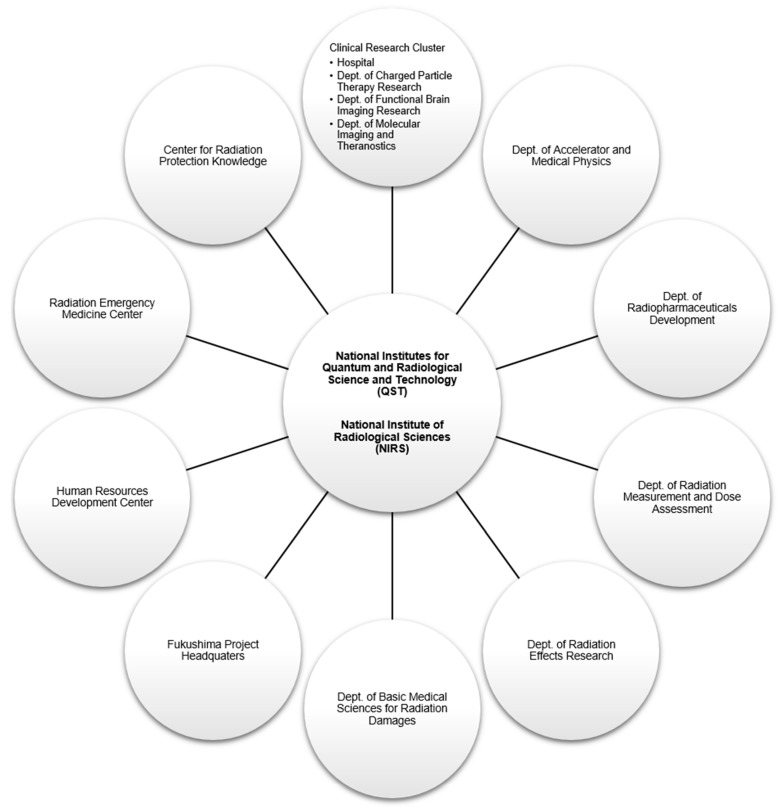
Organizational structure of the National Institute of Radiological Sciences. Other departments not included in the diagram: Research Planning and Promotion Office, Dept. of Administrative Services, Dept. of Engineering and Safety, and Quality Assurance and Audit Office.

The NIRS was established in 1957 with the mission to advance basic and applied research into radiological sciences in Japan. The current organizational structure of the NIRS is shown in Figure 1. In the center of its core mission, the hospital of charged particles has taken the initiative to develop world-class radiation therapy technology and to investigate the effects of radiation exposure on humans and the environment. Prior to adopting CIRT, hundreds of patients were treated with neutrons or protons at the NIRS since 1975 [8,9]. After extensive study of different ions for optimal physical and biological advantages, carbon particles were selected as the ion of choice in the HIMAC. Building on prior experiences with PT and the collaboration with the Lawrence Berkeley National Laboratory (LBNL) which has previously treated some patients with carbon particles in the 1970s, the decision to build the HIMAC was born in 1984 as part of a long-term cancer control plan in Japan. It took about 10 years before the first patient was treated in 1994. The cost of construction exceeded 32 billion Japanese Yen (~300 million USD in current estimates). Here is a brief history of the NIRS (excluding milestones unrelated to radiotherapy):

- NIRS was founded in 1957.
- Fast neutron therapy started in 1974 and proton therapy in 1979.
- HIMAC planning and construction started in 1984.
- HIMAC construction was completed at the end of 1993.
- HIMAC commissioning was completed and first patient was treated in June 1994. Only passive beam irradiation was available until 2011.
- Hospital for charged particle therapy opened in 1996–1997.
- CIRT was approved as advanced medical technology by the Japanese government Ministry of Health, Welfare and Labor in 2003. As such, the NIRS could receive reimbursement for CIRT. Cost was fixed per treatment independent of the number of fractions.
- Construction of the New Particle Therapy Research Facilities started in 2006.
- NIRS collaborated with Gunma University to construct a compact CIRT center with 1/3 the size and cost of the HIMAC at Gunma University in 2010.
- Active scanning treatment started in May 2011 in the new facility. A benchmark of 6000 patients were treated by 2011.
- Respiratory-gated phase-controlled rescanning irradiation of moving targets started in March 2015.
- A benchmark of 10,000 patients were treated by 2015 (one fourth were patients with prostate cancer).
- Construction of the superconducting rotating gantry was completed in 2015 [10].
- Commissioning of the gantry was completed in 2016 (half the size and weight of the gantry in the Heidelberg ion therapy (HIT) center in Germany).
- The NIRS has established a workflow system allowing the treatment of >800 patients annually.
- In April 2016, the National Institutes for Quantum and Radiological Science and Technology (QST) was established by merging the NIRS with the quantum beam and nuclear fusion departments of the Japan Atomic Energy Agency (JAEA).

## 3. Carbon Ion Radiotherapy in the Context of the Japanese Healthcare System

Japan has adopted a universal health coverage system since 1961. Almost all medical services are typically covered, with co-payments in the 10–30% range depending on patient’s age and income. When it opened in 1994, all patients were treated on clinical trials and the NIRS did not receive any reimbursement for these treatments. As results started to show the efficacy of CIRT, the Ministry of Health, Welfare and Labor declared CIRT as advanced medical technology in 2003. As such, the NIRS could be reimbursed for treating patients. However, the national health insurance did not reimburse those who wanted to receive CIRT. Since 2016, unresectable sarcoma became the only tumor type where CIRT is covered under the national healthcare insurance in Japan. The current available evidence suggests that, in addition to unresectable sarcoma, the superior benefits of CIRT are evident in patients with locally advanced pancreas cancer, recurrent rectal cancer, high-risk prostate cancer and non-squamous cell carcinomas of the head and neck. The Japanese healthcare reimbursement system is undergoing significant changes and a modification of CIRT reimbursement is expected in the near future with more cancer sites expected to be covered by the national insurance.

## 4. Carbon Ions as the Particles of Choice

Some definitions are important in order to explain the reasons for choosing carbon ions for therapy at the NIRS. Linear energy transfer (LET) represents the ionization density of a particular beam per unit track length. Relative biological effectiveness (RBE) is defined as the ratio of a reference radiation dose (typically photons of 250 kVp energy) to a test radiation dose in achieving the same biological endpoint under the same conditions. Oxygen enhancement ratio (OER) is defined as the dose of a particular radiation needed to result in an equivalent biological endpoint in the absence or presence of oxygen. In simplified terms, beams with high RBE and low OER are good candidates for particle therapy.

**Figure 2:**
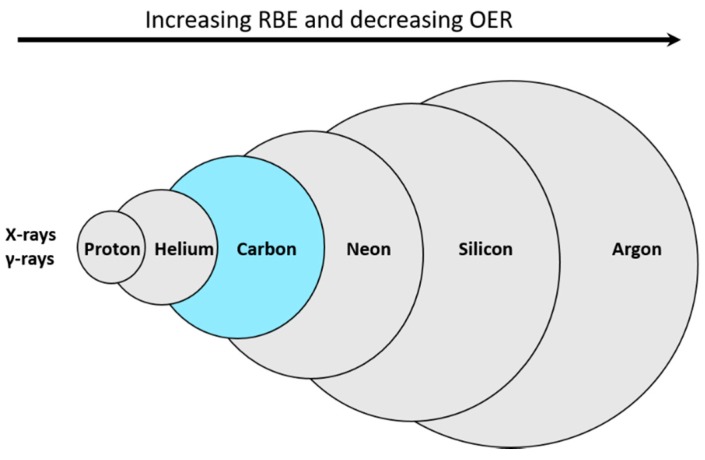
Simplified schematic diagram showing the relationship between atomic mass, relative biological effectiveness (RBE) and oxygen enhancement ratio (OER) for different particles in comparison to photons. The size of each circle represents atomic mass (not drawn to scale).

RBE typically increases with increasing LET until about 100 keV/µm, beyond which it plateaus or decreases [11]. OER, on the other hand, typically declines with increasing LET until it reaches 1. Light ions (such as protons) have low LET which may increase towards the end of the Bragg peak compared to heavier ions where the high LET dominates across the Bragg peak. As the atomic number increases further, LET increases. For very large particles like argon for example, the increase in LET occurs in the plateau region of the beam’s path risking an excessive normal tissue injury in radiation therapy. At the same time, the very high LET at the distal edge of the Bragg peak results in a sub-optimal RBE which reduces its clinical effect. While OER is most optimal (close to 1) with high LET ions, high LET may however cause unwarranted normal tissue damage. Accordingly, carbon ions have been considered to be a good compromise with an optimal RBE and OER and the best choice for treating cancer with PT [12] (Figure 2). Similar results were also reported by other international groups [11,13]. Additionally, another reason why carbon ions were chosen by the NIRS is that they have similar LET to neutrons which have been in clinical use at the NIRS for two decades prior to initiating CIRT [14]. In comparison to protons for example, carbon ions, with six times the charge and 12 times the mass, have 36 times higher LET for the same speed. In addition, carbon ions have reduced charge to mass ratio and thus sharper penumbra, but increased nuclear interactions and higher nuclear fragmentation beyond the Bragg peak.

## 5. Clinic Design

Building CIRT centers is costly and complicated [5]. Design of these facilities should take into consideration the treatment delivery method, number of rooms needed, choice of gantry (or not), and the expected number of fractions or patients to be treated annually. It is, of course, of the highest economic utility to treat the most number of patients, in the shortest time, with the least number of fractions with minimal set-up positions and immobilization devices, and shorter daily (in both broad and scanning beams) and patient-specific (in scanning beams) quality assurance (QA) times while maintaining highest quality beams and treatments. In designing the charged particle therapy facility at the NIRS, the goal has always been to adopt a patient-centered approach delivering the most potent, most accurate and most convenient regimens possible.

### 5.1. Heavy Ion Medical Accelerator in Chiba (HIMAC)

**Figure 3:**
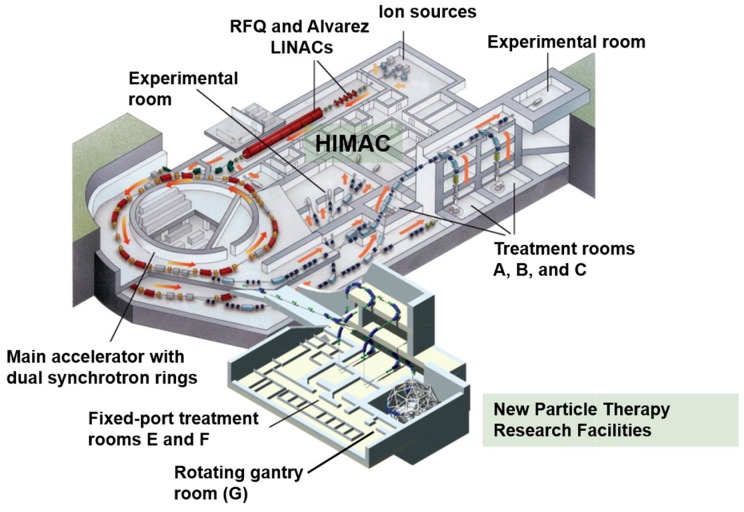
Overview of the HIMAC and New Particle Therapy Research Facilities at the National Institute of Radiological Sciences in Chiba, Japan.

The HIMAC is built on an area of about 120 × 65 m^2^ and houses the synchrotron which consists of ion sources, a linear accelerator cascade made of a radiofrequency quadrupole (RFQ) and an Alvarez linear accelerator (that can accelerate ions up to 6 MeV/u), dual synchrotron rings (which accelerate ions to 73% the speed of light), and independent horizontal and vertical high-energy transport beam lines which deliver the accelerated carbon ions to three treatment rooms with fixed ports: room A (vertical), room B (vertical and horizontal) and room C (horizontal) (Figure 3). In addition to these vertical and horizontal ports, the patient can be immobilized in the supine or prone positions with additional degrees of freedom provided by up to 20–30° tilt angle of the treatment couch. While these positions improve tumor targeting in fixed-port rooms, they add a significant workload during simulation and treatment. There are few other rooms for radiation physics and biology experiments in the HIMAC. The accelerated ions beams are currently extracted using the RF-knockout (RF-KO) slow extraction method [15]. This system allows a dynamic and precise control of beam intensity, position, on/off switching and transport to the downstream beam delivery systems. Interestingly, the HIMAC is capable of accelerating ions other than carbon, which is essential for the future development of new treatment strategies in charged PT (See Section 9).

### 5.2. New Particle Therapy Research Facilities

The New Particle Therapy Facility was designed to allow for the adaptive radiotherapy of tumors which are constantly changing in size, shape and location during treatments [16]. Thus, fast three-dimensional (3D) scanning was adopted as the treatment method of choice for the new facility (Table 1). The new facility uses the HIMAC upper synchrotron ring and houses three treatment rooms: rooms E and F have horizontal and vertical fixed ports (first patient was treated in May 2011) and room G has the state-of-the-art rotating gantry (Figure 3). Each room is equipped with a computer-based patient positioning system with orthogonal X-ray imagers and corresponding flat panel detectors. This positioning system allows excellent set-ups with minimal translational or rotational residual errors (<0.5 mm and 0.2°, respectively) [17]. While the HIMAC synchrotron will continue to support the new facility, the HIMAC will soon shut down its clinical operations and its treatment rooms will be limited to particle therapy research.

### 5.3. The World’s First Superconducting Rotating Gantry

**Figure 4:**
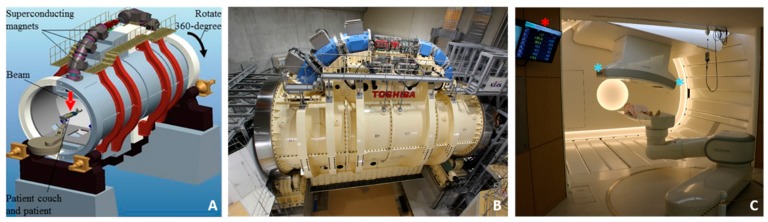
(**A**) Schematic diagram of the actual gantry seen in (**B**). Superconducting magnets, beam nozzle, and patient location are labelled. (**C**) A photograph showing a phantom patient on the robotic couch with the gantry in a slightly titled position. Also seen in the picture: the computer-based patient positioning system coordinates (red asterisk) and the flat panel detectors (blue asterisks).

The construction of the rotating gantry at the New Particle Therapy Research Facilities was completed towards the end of 2015 (Figure 4). It is the first PT gantry to use superconducting magnets, allowing a significant size and weight reduction (300 tons in weight and 13 m in length compared to 600 tons and 25 m at the HIT in Germany). These superconducting magnets rely on compact cryogenics to maintain critical temperatures for the zero electrical resistance states. The first patients were treated in 2017, and treating patients with moving targets started in January 2018. The gantry is able to irradiate patients at multiple angles, and as such, its use is expected to significantly reduce workload by eliminating the need for multiple set-ups, immobilization devices, and tilting couches. This will reduce treatment-related patient stress, increase treatment efficiency, and possibly improve efficacy and reduce normal tissue complications. Since the patient can be treated with different beam angles, treatments in the gantry room will also eliminate the need for deformable registration when patients are set-up in different positions, and this will eventually reduce uncertainty in dose calculations. The combination of the gantry system, the scanning irradiation and respiratory gating will give birth to state-of-the-art irradiation treatments.

The success with the use of superconducting magnet technologies in the NIRS gantry has opened the field of superconducting technology to the design and construction of smaller and cheaper synchrotrons. The SUPERconducting Magnet INstalled Ion Medical Accelerator in Chiba (Super MINIMAC, or the Quantum Knife) is the future vision of the NIRS in facility design with superconducting magnets in both the accelerator and gantry systems with significantly less cost (<50 million USD around year 2030) and a treatment capacity of 500 patients per year.

## 6. Treatment Delivery

### 6.1. Passive Beam Irradiation

Passive beam irradiation has been employed since 1994. The narrow “pencil” beam is received from the synchrotron rings by the beam delivery systems and is widened to create a uniform “broad beam” by the wobbler-scattering method. The wobbler magnets are located 9.9 and 11.7 m upstream of the isocenter in the vertical and horizontal ports, respectively. The wobbler magnets and scattering boards are able to generate different sizes of uniform irradiation fields with less loss compared to the double-scattering method. These beams are, however, mono-energetic and pristine. Ridge filters (aluminum and brass) are used to create spread out Bragg peaks (SOBP) of varying widths (maximum SOBP of 15 cm is able to cover most patients) by superimposing many pristine Bragg peaks. A range shifter (polymethyl metacrylate, PMMA) and patient-specific compensator (polyethylene) are used to conform the depth of the SOBP to the distal edge of the tumor. Multi-leaf collimators (MLC) and patient-specific (brass) collimators are used to cut the lateral beam edges to the shape of the tumor. Patient-specific collimators and compensators are individualized for each patient and for every irradiation direction, thus increase treatment cost and delay treatment start. Passive irradiation increases neutron scattering and integral dose [18,19], has an excess dose proximal to target tumors, and low beam utilization efficiency (10–30%). On the other hand, passive irradiation is insensitive to target motion. To deal with the excess proximal dose, the layer stacking method has been developed [20]. Using the same delivery system, thin (usually 1 cm) Bragg peaks are stacked in the distal to proximal direction by changing the range shifters to control depth while modulating the MLCs to conform to tumor shape at each depth.

### 6.2. Three-Dimensional Scanning Irradiation

In contrast to passive techniques, scanning irradiation utilizes beams with narrow Bragg peaks created using mini ridge filters (two types, PMMA or aluminum) to “paint” dose continuously over tumors. Transverse scanning is modulated by two scanning magnets. The distance from the first magnet to the isocenter is 8.4 m. Depth, on the other hand, has been modulated with different technologies over the past few years. Initial scanning technology in 2011 used range shifters for depth modulation. The use of range shifters, however, increased lateral beam size and neutron contamination. In 2012, a hybrid system of energy modulation and range shifters was implemented [21]. Full energy modulation scanning has been in use since 2014 by a multiple-energy operation with extended flattops of the synchrotron [22]. As such, various energies (highest energy first followed by lower energies) can be extracted during the extended flattops and multiple depths are treated (distally to proximally) within a single synchrotron spill without the need for energy degraders (such range shifters). Two hundred energy steps are currently available. This allows irradiating complex tumor shapes with less dose spillage into surrounding critical structures. Full energy modulation eliminates the need for range shifters and patient-specific compensators and collimators, thus reducing the lag time to treatment and cost of patient set-up. While the hardware and work-load are reduced with scanning irradiation, this delivery system needs to precisely control beam size, beam position and particle fluence at each irradiation spot for an optimal dose distribution as calculated by the treatment planning system. This requires significant engineering and synchronization technologies between the synchrotron and the treatment delivery system. Any error in beam size, position and fluence should be immediately detected by the multiple beam monitors to shut off the beam. In the absence of beam barriers, scanning irradiation reduces proximal dose excess, improves beam efficiency (almost a 100% beam efficiency compared to 10–30% with passive CIRT), and reduces range loss. In passive beams for example, water-equivalent depth is 25 cm for 400 MeV/u energy compared to 27 cm for scanning beams. In theory, since it provides a more flexible target shaping, it may also improve local control with better dose-escalation capabilities, reduce treatment-related adverse events, and improve patients’ quality of life. This, however, still needs to be verified prospectively. Taken together, scanning beams will allow an easier adaptive CIRT.

Scanning irradiation was initially limited to static tumors because it is extremely sensitive to target motion and, thus, elaborate motion management schemes are needed for thoraco-abdominal tumors. To treat moving tumors, the NIRS had to develop an extremely fast scanning magnet, while at the same time maintain the ability to monitor beam quality and control scanning speeds. The NIRS has developed the world’s fastest phase-controlled 3D scanning irradiation system (up to 100 mm/ms (V_x_) and 50 mm/ms (V_y_), at the isocenter, up to 30 cm deep at 430 MeV/u) [23,24,25] which has successfully expanded CIRT application to moving tumors with reasonable treatment times [17,26].

### 6.3. Motion Management

While motion management is important in all radiation treatments, it acquires a special significance in CIRT given the sharp distal fall off after the Bragg peak. Indeed, a 1 cm shift may cause significant dose difference in CIRT compared to an insignificant difference in photon radiation. In addition to set-up variations and inter-fractional motion, target shifts are governed by intra-fractional tumor motion. For this review, we will focus on the efforts within the NIRS to manage irradiation in tumors with intra-fractional motion. Thoracic, abdominal and pelvic tumors are affected by respiration, heart beats, and peristalsis to varying degrees. The NIRS has performed extensive studies on the intra-fractional motion of tumors in these locations and accordingly developed techniques to mitigate the effect of such motion on treatment delivery [27,28,29].

Prior to scanning irradiation, respiratory gating was used for target motion management [30]. Nearly half of all patients treated at the NIRS have benefited from respiratory gating technologies including lung, pancreas, liver, rectal, uterine cancers, and occasionally sarcomas. Respiratory phases are detected by tracking the motion of the chest wall/abdomen surface, and CT simulation scans are synchronized with the respiratory motion. Respiratory-gated simulation, planning, patient set-up, and irradiation are synchronized with the gating signal (using peak exhalation as the gating window). Gating is imperfect, however, due to an unsatisfactory reproducibility of the respiratory phases and a slight residual motion in the target tumor. This led to significant aberrations in dose distribution within the target with scanned beams (this is called the interplay effect; a good visual display is seen in Figure 8 in [31]). Thus, respiratory gating alone was not enough for motion management in scanning CIRT. One way to minimize the interplay effect is a combination of gating, fast phase-controlled rescanning, and range-ITV (internal target volume) based treatment planning [32]. Phase-controlled rescanning is the technology that allows fast re-irradiation of each point in the tumor multiple times during each fraction to suppress hot/cold spots. Such a system requires a near-perfect coordination between the accelerator, the scanning magnets, and the monitoring/feedback systems. Range-ITV is a margin technique in treatment planning which accounts for intra-fractional range uncertainties by calculating the minimum and maximum beam range along each beam using data from 4D-CT (four dimensional CT) scans. This approach has expanded the utility of scanned beam CIRT by providing conformal and homogeneous plans for moving targets in manageable treatment times.

Currently, markerless tracking, using deep learning networks in image processing, can track tumors without the need of fiducial markers [33]. Markerless respiratory-gated CIRT has been developed for moving tumors [34,35]. In this technology, the position of the moving tumor can be calculated in real time using X-ray fluoroscopy (with the use of X-ray sources and dynamic flat panel detectors in the treatment room), and the scanning beam is “on” only when the center of mass of the tumor relocates to a predefined area in accordance with the respiratory cycle. In March 2015, the NIRS treated the first lung cancer patient using respiratory-gated fast rescanning CIRT [36]. Excellent reviews of the NIRS pencil beam scanning system [31] and motion management technologies [37] have been recently published and are worth reading for physicians or physicists interested in a more detailed explanation.

## 7. Dose Prescription and Treatment Planning

The NIRS has realized the importance of accurate treatment planning in CIRT and has developed its own software (HIPLAN) [38]. The goal has always been to calculate dose deposited in targets, and dose absorbed in normal surrounding tissue. In addition, the system had to account for the energy spectra of the projectile fragments and their pathways. This complexity led to the development of a rather simplified but dependable system with simplified parameters.

The method of dose prescription at the NIRS has been previously described [14,39,40] and updated [41]. We will here emphasize the major points. Dose in CIRT plans is usually presented as RBE-weighted absorbed dose, unless otherwise specified. This RBE-weighted dose (unit is Gy RBE) is the product of deposited physical dose of the carbon beams and the assumed RBE value. RBE, however, is a complex measurement usually influenced by LET, target depth, tumor type, dose, fractionation, oxygenation, and the selected endpoint, among other variables. Given its dependence on many variables, it is difficult to accurately determine RBE, and thus, for treatment planning, a clinical RBE is assumed rather than proven. It is difficult to estimate clinical RBE by comparing clinical results of CIRT to photon irradiation due to the different fractionation schemes used in the different trials.

The first patients expected to receive CIRT at the NIRS were patients with salivary gland tumors, and thus the initial dose prescription method built on results using in vitro data of human salivary gland (HSG) cell survival curves and the NIRS experience using fast neutrons in the clinic. Later experiments showed little variability in RBE of carbon ions among different cell lines [42]. The following explanation describes the process in a stepwise fashion:

- **Step 1, Determining biological RBE**: To create an SOBP with uniform cell kill, the linear-quadratic model variables α and β had to be determined. Cell survival curves were initially created for HSG cells using multiple monoenergetic pristine carbon ion beams and the corresponding α and β values were calculated as a function of depth. An SOBP, however, is characterized by a distribution of LET values. Consequently, α′ and β′ values were determined for a mixed beam of multiple Bragg peaks. Accordingly, survival (10% survival) of HSG cells could be determined at any depth in a given SOBP. This allowed the creation of a ridge filter that could achieve a uniform cell kill effect of HSG cells along the SOBP. Because LET generally increases towards the end of the SOBP, the ridge filter design reduces the weight of the physical dose with depth. This was tested experimentally by irradiating HSG cells using a 6 cm SOBP of 290 MeV/u carbon ion beams, and the results allowed the determination of the biological RBE and RBE-weighted dose distribution [43]. Clearly, this initial model did not account for dose level in RBE calculation.
- **Step 2, Determining the neutron equivalent point**: The NIRS has a long history using fast neutrons in the clinic since 1974. The NIRS team aimed to find a depth in the carbon beam SOBP at which neutrons would exhibit the same RBE. Using an average of in vitro (again 10% survival of HSG cells) and in vivo (mouse skin dry desquamation) data, it was decided that the depth corresponding to an LET value of 80 keV/µm, 8 mm upstream of the distal fall off of a 6 cm SOBP of carbon ion beams of 290 MeV/u, to be the neutron equivalent point. At this point, it was thought that neutron clinical RBE would be equivalent to carbon ion clinical RBE.
- **Step 3, Determining clinical RBE**: The NIRS team decided to initially use a similar fractionation schedule for CIRT as was being used for neutrons. The neutron clinical RBE has been previously determined to be 3.3 for a 20-fraction treatment and thus, an RBE of 3 for an 18-fraction regimen was selected. The flat top of the biological dose is first determined by the treating physician. Using a clinical RBE of 3, the physical carbon ion dose is determined at the neutron equivalent point. The physical dose distribution is then normalized to the neutron equivalent point which allows the determination of the physical dose at the center of SOBP. The RBE values at the center of the SOBP are obtained by dividing the biological dose by the physical dose. A table of clinical RBEs at the center of the SOBP is created for each SOBP width.
- **Step 4, Updating the biophysical model**: Given the lack of reliable experimental evidence in 1994, some assumptions had to be made to simplify the above biophysical model. LET was assumed to be an accurate predictor of RBE. In reality, LET does not fully explain the distribution of energy deposition around a particle track and RBE is also dependent on other variables such as, but not limited to, tissue type, fractionation and dose level. With the accumulated knowledge, it was necessary to update the biophysical model to account for the improved understanding of the mechanisms of biological effects of carbon beams. Hence, the Microdosimetric Kinetic Model (MKM) [44], which attempts to explain the biological effects of irradiation beams based on the stochastic energy deposition of carbon ions at the micrometer level, was adopted in the updated version of the treatment planning system used for scanning irradiation [45,46]. Comparative experimentation showed that the original model was adequate for clinical use [47] but the updated model better estimated dose distribution especially at the tail beyond the distal fall off where nuclear fragments are in excess [41]. Variable-RBE models are necessary for an utmost utilization of the advantages of CIRT. Given the inadequacy of RBE for the ideal treatment planning system especially for extreme hypofractionation, alternative parameters for plan optimization and evaluation are being considered to decrease the reliance on RBE. The NIRS continues to improve its dose calculation models [48]. A different approach to dose prescription has been proposed, utilized and updated for treatment planning at the Gesellshaft für Schwerionenforschung (GSI) in Germany: namely the Local Effect Model (LEM) [49,50,51,52]. Efforts between the NIRS and the European centers are underway to better understand and minimize the differences between the two models [53,54].

For every treatment to be realized, the radiation oncologist and dosimetrist need to make important decisions about beam number and beam angles. For patients not treated in the gantry room, beam arrangement should be decided prior to CT simulation since patients need to be set-up accounting for beam angles and couch tilts. In addition to beam arrangements, other decisions need to be made regarding uncertainty margins and optimization algorithms. Similar to photon therapy, treatment plans should account for set-up errors and inter- and intra-fractional target motion using planning target volume (PTV) expansion. In addition however, CIRT planning needs to account for range uncertainty and thus another margin needs to be added in the longitudinal (depth) direction of each beam. Thus, for every treatment plan in CIRT, an initial PTV is added isotropically for set-up uncertainty and an additional field-specific margin is added for each beam for range uncertainties. The task of selecting the latter is more cumbersome for moving targets and a field-specific treatment volume (FTV) is created. In determining the optimal number of particles for each beam (i.e., plan optimization), the dosimetrist aims for a homogeneous dose in each beam using Single Field Uniform Dose (SFUD), or a non-uniform dose in individual beams whose sum will bring a uniform overall plan such as in Intensity Modulated Particle Therapy (IMPT). Only a limited number of patients have been treated with IMPT at the NIRS so far.

## 8. Hypofractionation in Carbon Ion Radiotherapy

The initial dose-escalation phase I/II clinical trials conducted at the NIRS aimed to establish the safety of CIRT, and to achieve local control for rare malignancies and for common cancers with poor outcomes with conventional treatments. In the first year between June 1994 and August 1995, a total of 55 patients were treated for various indications: head and neck (14), brain (10), lung (13), liver (5), prostate (2), cervix (3), and others (8). Subsequently, clinical trials for bone and soft tissue sarcomas started in 1996, renal cell carcinoma in 1997, pancreas cancer and recurrent rectal cancer in 2000, uveal melanoma in 2001, and esophageal cancer in 2004. The number of patients treated at the NIRS per indication is shown in Table 2.

From a clinical point of view, the high LET of CIRT improves the peak-to-plateau ratio, thus allowing a higher chance of tumor cell kill/control while maintaining a low risk of normal tissue complications. The average number of fractions per treatment at the NIRS was 18 in 1995 but it dropped quickly to become 12 fractions in 2016 (data averaged from 11,249 treatments). Most of the drop occurred before the year 2000. With hypofractionated regimens, the NIRS can manage more patients and the cost effectiveness for CIRT can be established. Moreover, patients, who travel to the NIRS from all over Japan and neighboring countries, will be more satisfied as they require fewer trips to finish their treatment. For intractable radio-resistant tumors (such as non-squamous cell carcinomas of the head and neck, skull base cancers, post-operative recurrent rectal cancer, unresectable sarcomas, and in cases of tumors recurrent after photon radiation), CIRT has been applied in 16 fractions over 4 weeks. Conversely, some common cancers (such as early stage lung cancer, and hepatocellular carcinomas) have been irradiated in 1–2 fractions. Other common cancers such as stage I esophageal cancer, pancreatic cancer and prostate cancer were treated in 8–12 fractions (see Table 3).

### 8.1. Physical and Biological Rationale of Hypofractionation in Carbon Ion Radiotherapy

A detailed explanation of the physical and radiobiological advantages of CIRT has been previously discussed in details [3,90]. In brief, carbon beams have improved dose distribution over photons due to their characteristic Bragg peak. Given the reduced charge-to-mass ratio, carbon ion beams also have reduced lateral scattering compared to proton irradiation. These characteristics allow the delivery of high doses per fraction to tumors while simultaneously sparing surrounding normal tissue due to low entrance dose and low entrance LET [1,2]. Moreover, CIRT has stronger biological effects compared to photon or proton radiotherapy due to their inherent ability to produce complex or clustered DNA damage which is refractory to repair. The effects of high LET carbon ion beams are also less dependent on molecular oxygen. Thus, for the high LET ranges, OER for CIRT can be as low as 1–2 indicating that carbon beams are more efficacious at killing cancer cells in hypoxic niches [91]. These characteristics are rather simplified in this brief description and are different for different particles even if they exhibit the same LET because the profiles of beam energy deposition along track structures is different for different ions, and hence the biologic effects are consequently different.

The differential response of tumors and normal tissue to photon radiation is best explained using the classic “4R” biological factors (repair, reassortment, repopulation and reoxygenation). Repair, reassortment, and reoxygenation improve the therapeutic ratio in fractionated treatments whereas repopulation does the opposite. These factors are more important in low-LET radiation (such as photon radiotherapy or the low LET entrance region of CIRT) but less important in the high-LET regions. Thus, a theoretical framework can be hypothesized explaining the benefits of using hypofractionation in CIRT based on these factors acknowledging that research on these factors in the context of CIRT is weak. Sub-lethal damage repair is expected in the normal tissue low-LET regions with CIRT compared to the high-LET tumor region which is strategically located within the Bragg peak. Additionally, unlike photons where cells are more sensitive in the M/G2 phases, carbon ions are able to induce tumor kill independent of the cell cycle phase [92,93]. Given the lower DNA repair capacity, less dependence on cell cycle, low OER, interest in CIRT hypofractionation peaked. Moreover, experiments and clinical experience with CIRT revealed that RBE decreases with higher doses per fraction [94]. This RBE change, however, is steeper for normal tissues (generally lower α/β) compared to malignant tissues [95]. Accordingly, hypofractionated CIRT increases therapeutic ratio.

### 8.2. Economic Rationale of Hypofractionation

To date, there are no reported results comparing carbon to proton or photon therapy to support the wide-spread expansion of CIRT centers. In theory and in most reported phase I/II trials, CIRT has shown an advantage over historical controls with photon irradiation either due to improved tumor control (such as in chordoma) or reduced adverse events (such as in high-risk prostate cancer). Despite this however, the high cost of constructing and maintaining CIRT centers remain the major barrier for their widespread adoption especially in the absence of high level cost effectiveness data and the absence of reimbursement models in the United States, for example. Very few studies evaluated the cost effectiveness of CIRT, although the initial verdict support the economic rationale at least in chordomas, recurrent rectal cancer and possibly non-small cell lung cancer [6,7,96,97]. Similar to the global situation, health care budgets in Japan are under extreme scrutiny and there is a significant new governmental pressure for adopting more cost effective treatments. Adopting a hypofractionated philosophy in approaching clinical trial design, whenever possible, maybe the most reliable method of enhancing cost-effectiveness of CIRT [5,98]. Even in Japan where the current reimbursement is per treatment rather than per fraction, hypofractionation will allow the treatment of more patients and an optimal utilization of resources.

## 9. Ongoing and Future Projects at the NIRS

In addition to the ongoing efforts in hypofractionation, dose escalation and the ongoing testing of the scanning irradiation and gantry treatments, several improvement projects are either ongoing or being planned. The following topics summarize some subjects of current clinical/preclinical research at the NIRS.

### 9.1. Combination with Systemic Therapy

Given the increased biological effectiveness of CIRT, concurrent systemic therapies are not needed for radio-sensitization. However, in some cancers with high metastatic potential such as pancreas cancer or mucosal melanoma, concurrent chemotherapy is now a standard adjunct to CIRT to reduce the risk of distant failures [78,99]. While cisplatin is also becoming standard concurrent therapy with CIRT in cervical cancer (including both squamous cell carcinoma and adenocarcinoma; data pending publication), only few other cases combine systemic therapy with CIRT [100,101]. While preclinical studies have generally demonstrated safe combination with chemotherapy [102,103,104,105,106,107,108,109], investigators at the NIRS have been extremely careful in proceeding with such therapies unless absolutely necessary such as the above mentioned cases. A current trial is investigating preoperative CIRT and concurrent cisplatin and 5-FU in esophageal cancer. On the other hand, combining CIRT with immunotherapy such as immune checkpoint inhibitors have not taken off except for a few patients who received CIRT after being on immune therapies without any alarming toxicity (unpublished data). While multiple preclinical studies have shown that CIRT is a stronger stimulator of the immune system [110,111], such combinations are still not planned for clinical trials at least pending some major animal experiments currently underway.

### 9.2. LET Painting and Mixed Beams Irradiation

One major indicator of cancer treatment resistance is hypoxia. Conventional photon fractionation allows for reoxygenation, but local relapses due to hypoxia continue to be a major cause of treatment failure. Low OER CIRT is an excellent candidate for treating hypoxic tumors. Unfortunately, however, OER is not 1 with CIRT. Using imaging techniques to delineate hypoxic regions [112], LET painting, which is similar conceptually to dose painting in photon therapy, is a biologically-driven technique to deliver a heterogeneous LET distribution such that high LET is painted to radiation-resistant (hypoxic) volumes and low LET is sequestered into normal tissue. The utility of LET-painting (and/or dose-painting) in these conditions is still experimental and patients have not yet reaped its benefits [113,114]. The NIRS is currently investigating methods for LET painting with mixed beams using a variety of potential candidate ions (such as Helium and Oxygen) that can possibly be accelerated in the HIMAC synchrotron. The actual implementation of this goal, however, still requires several technological advances in particle accelerators (multi-ion capabilities with fast switching, for example) and treatment planning.

### 9.3. Artificial Intelligence

Radiation oncology is a data-driven field with unique dependence on technology and statistical modeling. Hence, there is a special interest in using artificial intelligence and machine learning techniques to promote research in radiation oncology [115]. Given the complexity of the physical, chemical and biological response to carbon ion track structures, artificial intelligence and deep learning networks may represent a solution for problems that require massive computational powers and complex modeling. Currently, the NIRS uses deep neural network-based real-time image processing for its image guided CIRT for scanning irradiation of moving targets such as lung tumors [33]. However, significant other avenues could benefit from this technology including target segmentation [116], range verification, dose distribution and treatment planning. Huge volumes of structured data are needed for this work but the NIRS is determined to explore this avenue.

### 9.4. Local and International Collaborations

The NIRS is invested in developing and promoting CIRT in Japan and globally. Successive CIRT centers have built on technological advances from the NIRS with special emphasis on reducing size and cost. Gunma University, for example, has installed its own accelerator in collaboration with the NIRS with one third the size of the HIMAC while maintaining comparable capability. Hyogo Ion Beam Medical Center (HIBAC) in Hyogo since 2002, Gunma Heavy Ion Medical Center (GHMC) in Gunma since 2010 [117], Saga Heavy Ion Medical Accelerator (Saga-HIMAT) in Tosu since 2013, and Ion-beam Radiation Oncology Center in Kanagawa (i-ROCK) in Kanagawa since 2015 are currently treating patients. The Osaka (expected to treat its first patient in 2018) and Yamagata (expected to treat the first patient in 2020) carbon ion centers are in the construction/planning phases. The Yamagata CIRT center will host a more compact gantry and scanning systems developed in collaboration with the NIRS. The active institutions collectively form the Japan Carbon-ion Radiation Oncology Study Group (J-CROS) which was established in 2014. The role of J-CROS is to enhance collaboration among the Japanese carbon treatment centers, develop technologies for PT, conduct multi-institutional clinical trials, and unify treatment approaches.

The NIRS has also become an international research institution where hundreds of distinguished researchers and clinicians from all over the world have visited, and many even stayed for months or years developing projects. Moreover, the NIRS allows its international collaborators to use carbon beams for their experiments. The NIRS aims to establish international groups for collaboration on trans-national clinical trials. The CIPHER trial, comparing IMRT to CIRT for locally advanced pancreatic cancer, is an example of remarkable collaboration efforts between the NIRS, the department of radiation oncology at the University of Texas Southwestern Medical Center in the United States, the Centro Nazionale di Adroterapia Oncologica (CNAO) in Italy, Peking Union Medical College in China, and the CIRT project of Yonsei University Health System in South Korea.

## 10. Conclusions

In 2015, the Lancet Oncology published a series of recommendations to advance CIRT after a panel of Japanese and international radiation oncologists, radiobiologists and medical physicists performed a complete review of the NIRS [118]. A summary of the recommendations and current status of work is reviewed in Table 4. 

The NIRS continues to strive to provide the best patient-centered care for individuals with rare and common cancers in Japan, and to disseminate the accumulated knowledge in accelerator technology and radiation treatments locally across Japan and globally. Optimization of treatment regimens will continue to progress as the NIRS steps into its second generation mode of operation and technologies and as it develops more compact and cheaper accelerators/gantries with improved specifications. Within the National Institutes for Quantum and Radiological Science and Technology (QST), the NIRS will integrate quantum technologies into its core mission and thus help incorporate quantum applications in CIRT.

## Figures and Tables

**Table 1:** Major specifications of the new particle therapy research facilities.

Specification	Value
Treatment rooms	Rooms E and F: H and V beams in each
	Room G: Rotating gantry (26 possible angles)
Accelerated energies	140–430 MeV/u
Range	Up to 30 cm
Field size	22 × 22 cm in rooms E and F
	20 × 20 cm in room G (compared to 15 × 15 cm in the HIMAC)
Dose rate	Up to 5 GyE/min
Irradiation method	3D fast rescanning with gating for moving targets
Scanning technology	Multiple-energy operation with extended flattops with >200 energy steps

Abbreviations: H, horizontal; V, vertical; HIMAC, Heavy Ion Medical Accelerator in Chiba; 3D, three dimensional.

**Table 2:** Number of patients per cancer site treated with carbon ions at the National Institute of Radiological Sciences between June 1994 and July 2017.

Site	Number (%)
Prostate	2863 (24.7%)
Bone & soft tissue	1336 (11.5%)
Head & neck	1107 (9.6%)
Lung	1062 (9.2%)
Pancreas	624 (5.4%)
Liver	613 (5.3%)
Rectum (post-operative relapse)	572 (4.9%)
Uterus (cervix & body)	289 (2.5%)
Uveal melanoma	206 (1.8%)
Abdominal lymph nodes	143 (1.2%)
CNS	106 (0.9%)
Skull base	104 (0.9%)
Gastrointestinal tract	97 (0.8%)
Lacrimal Gland	37 (0.3%)
Scanning beams (clinical trial)	21 (0.2%)
Breast	9 (0.1%)
Kidney	8 (0.1%)
Rotating gantry (clinical trial)	8 (0.1%)
Re-irradiation	1065 (9.2%)
Others	1310 (11.3%)
Total	11,580 (100%)

**Table 3:** Summary of initial and current fractionation schedules for the most common cancers treated at the National Institute of Radiological Sciences.

Cancer Type	Number of Fractions in Initial Protocols	Current Clinical Practice (Shortest Regimen)
Osteosarcoma and Soft Tissue Sarcoma [55,56,57,58,59,60]	16	16
Head and neck cancer ^a^ [61,62,63,64]	18	16
Lacrimal gland tumor [65]	12	12
Ocular melanoma [66,67]	5	4
Skull base cancer [68,69,70,71]	16	16
Early stage lung cancer [47,72]	18	1
Hepatocellular carcinoma [73,74,75,76,77]	15	2
Liver metastasis from colorectal cancer ^b^	1	1
Pancreas cancer [78,79,80]		
-Preoperative	16	8
-Definitive ^c^	12	12
Esophageal cancer [81]		
-Preoperative ^d^	8	8
-Definitive	12	12
Recurrent rectal cancer [82]	16	16
Renal cell carcinoma [83]	16	4 (on protocol)
Prostate cancer ^e^ [84,85,86]	20	12
Locally advanced uterine (cervical) cancer ^f^ [87,88,89]	24	20

^a^ Mostly for non-squamous cell carcinomas (adenoid cystic carcinoma, adenocarcinoma, and mucosal malignant melanoma). Treatment of melanoma uses concurrent chemotherapy. ^b^ Unpublished data, paper in submission. ^c^ Concurrent gemcitabine is standard treatment with CIRT for locally advanced pancreatic cancer. Future trials will focus on dose escalation, LET-painting or mixed beam irradiation rather than further hypofractionation. ^d^ Current trial is testing preoperative carbon ion therapy (8 fractions) and concurrent cisplatin and 5-FU. ^e^ Future clinical trials may test 8 fractions in 2 weeks; not in effect yet. ^f^ Uterine cancer include both adenocarcinoma and squamous cell carcinoma. Weekly concurrent cisplatin is now standard.

**Table 4:** A summary of recommendations of international experts to the NIRS and current progress.

Recommendation	Progress
*Clinical*	
“Continued research in ultra-short fractionation”	Significant milestones achieved (single fraction NSCLC) and multiple clinical trials in progress
“Continued research in combined modalities”	Ongoing work in pancreas, melanoma, uterine, and esophageal cancers
“Reduction in size and cost of technology”	Significant milestones achieved (Section 9.4) and more work in progress
“Improving patient throughput including use of gantry, immobilization devices”	Patients, including those with moving tumors, are currently being treated in the gantry room
“Analyzing incidence of SMN after CIRT”	Ongoing work
“Publish studies in peer reviewed journals and provide detailed reporting of methods in studies”	Tens of papers have been published with updated reporting of results and methodology
“Increase use of QOL assessment”	QOL studies are in progress [119]
“Announce and register clinical trials internationally”	Clinical trials are announced online at www.umin.ac.jp/ctr
“Analysis of relations between dose and local recurrence to estimate potential of dose-painting”	Ongoing work
“Start scanning beams CIRT for moving targets”	Patients with moving tumors are treated regularly with scanning beams
“Use MRI for adaptive therapy for cervical cancer”	MRI is now standard practice for adaptive planning in cervical cancer
“Start randomized phase III trials”	CIPHER trial will start accruing soon (Section 9.4)
“Start trials on GBM”	No ongoing trials at the NIRS for GBM
*Radiobiology*	
“Intensify international collaborations and harmonize reporting of data/methods”	Ongoing work
“Achieve international standard for biophysical modeling in treatment planning”	Ongoing work with European teams [54]
“Establish dose-dependent RBE especially for hypofractionation”	Ongoing work [48]
“Continue work on combinations of CIRT and immunotherapy”	Ongoing preclinical studies
*Medical physics*	
“Continue commissioning of moving target irradiation, PCR and tumor tracking gating system” and “continue research on the interplay effect of scanning beams”	Patients with moving tumors are treated regularly using PCR and respiratory gating
“Continue work on optimization of multiple energy operations of synchrotrons”	Significant progress achieved [22]
“Continue work on gantry commissioning”	Patients, including those with moving tumors, are currently being treated in the gantry room

Abbreviations: NIRS, National Institute of Radiological Sciences; NSCLC, non-small cell lung cancer; SMN, second malignant neoplasms; CIRT, carbon ion radiotherapy; QOL, quality of life; MRI, magnetic resonance imaging; GBM, Glioblastoma Multiforme; RBE, relative biological effectiveness; PCR, phase-controlled rescanning.
